# Supplementary figures and images for: Protein degradation sets the fraction of active ribosomes at vanishing growth
Source: PLoS Comput Biol. 2022 May 2;18(5):e1010059. doi: 10.1371/journal.pcbi.1010059 (PMC9098079; doi:10.1371/journal.pcbi.1010059)

**(a)**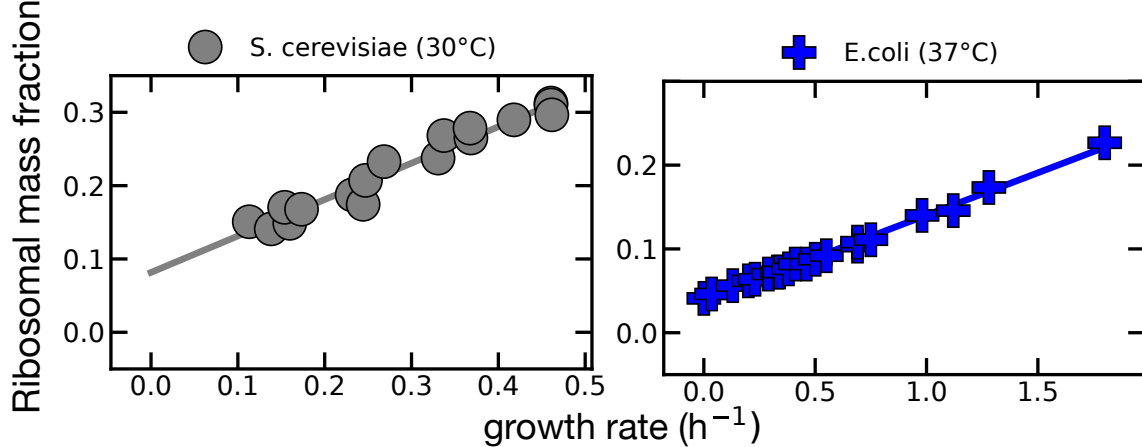**(b)**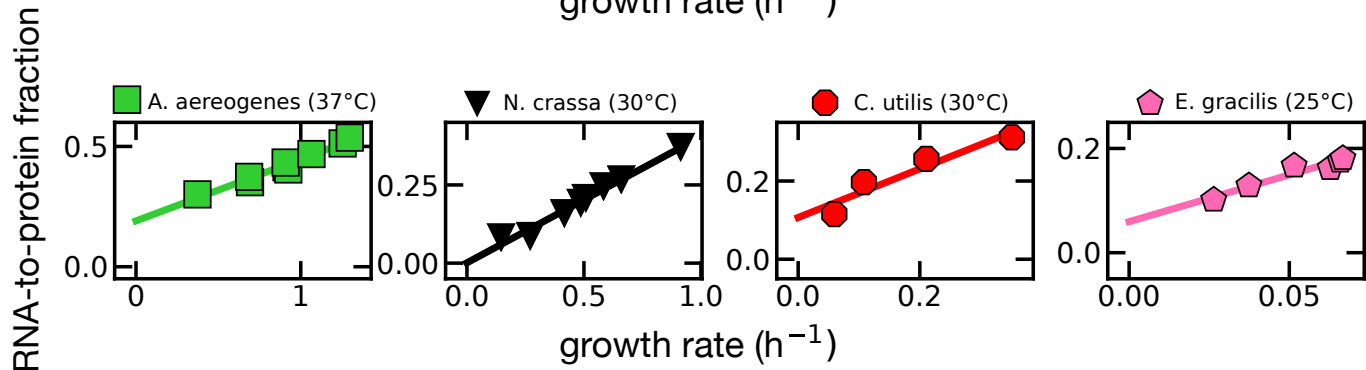

Supplement: S1 Fig — (a) Data on ribosomal mass fraction for E. coli and S. cerevisiae. (b) Data on RNA/protein ratios for other organisms. Data from [4] (S. cerevisiae), [5] (E. coli), [52] (A. aerogenes), [53] (N.crassa), [54] (C. utilis), [55] (E. gracilis). (PDF) [file pcbi.1010059.s001.pdf]

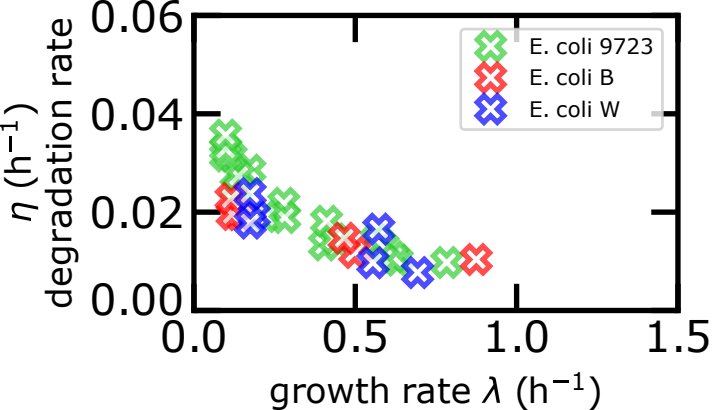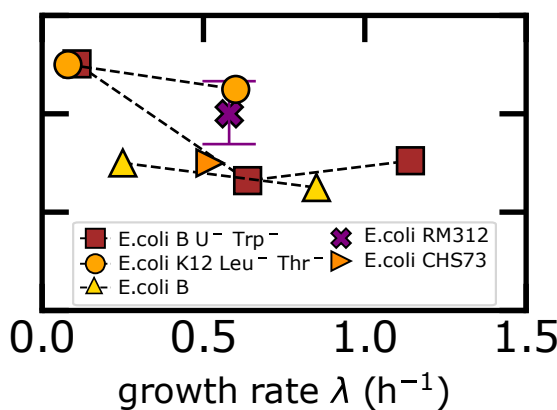

Supplement: S2 Fig — (a) Degradation rate across growth conditions from [38] as used in the main text. (b) Degradation rate across different growth conditions from other studies using different strains and techniques, [36] (E. coli B), [39] (E. coli RM132), [40] (E. coli CHS73), [57] (E. coli B U−1 Trp−1), [58] (E. coli K12 Leu−1 Thr−1). (PDF) [file pcbi.1010059.s002.pdf]

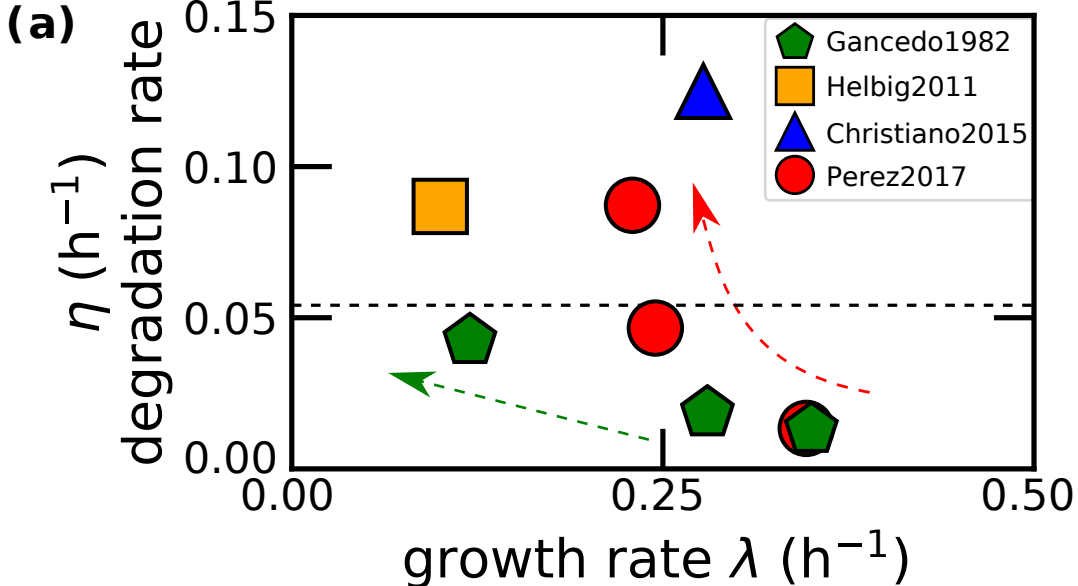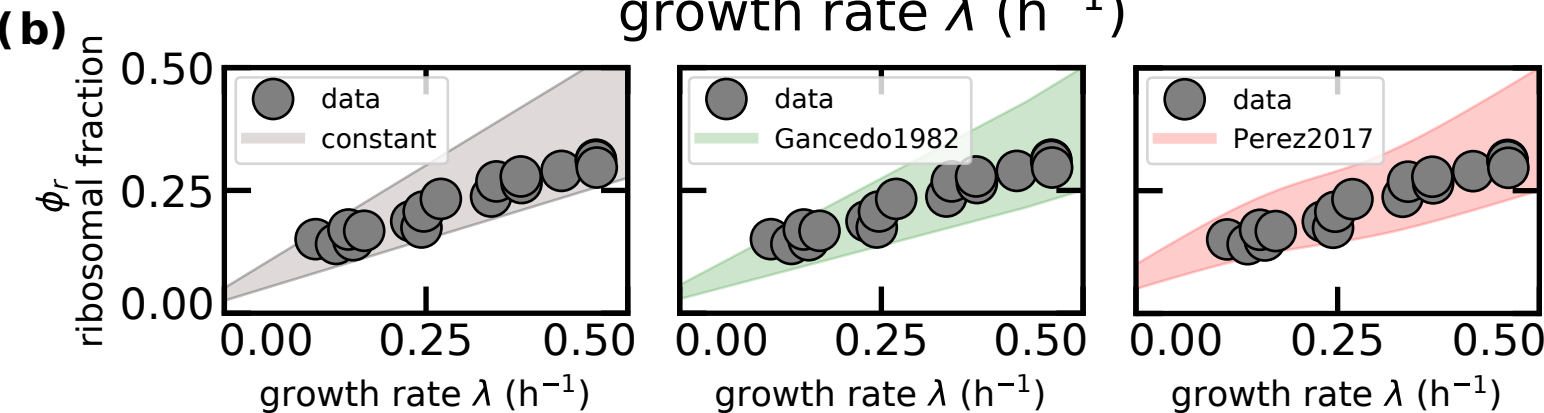

Supplement: S3 Fig — (a) Mean degradation rate across growth conditions from [23, 42, 46, 48] respectively using strains CJM13, CEN.PK113–7D DBY10144 and BY4742. The dashed line indicates the average of all the shown points (which are averages in a single condition). The dashed arrow lines highlight the increasing trends of degradation rates with decreasing growth rates in two data sets. (b) The range of predicted ribosomal fractions of the model, plotted next to data points from [4] that uses strain BY4742. The model requires as inputs degradation rates and translation elongation rates. As a value for the degradation rate, we have taken the mean value, shown in panel a as a dashed line in the left subpanel, as well as a linear fit of the degradation rate from [42] (green hexagons in panel a, central subpanel in panel b), and a sigmoid fit for [23] (red circles in panel a, right-hand subpanel in panel b), see also Methods and Materials. We then considered a range of physiologically relevant translation elongation rates (3–8 aa s−1) from ref. [51]. The shaded area represents the prediction of the model for such range. (PDF) [file pcbi.1010059.s003.pdf]

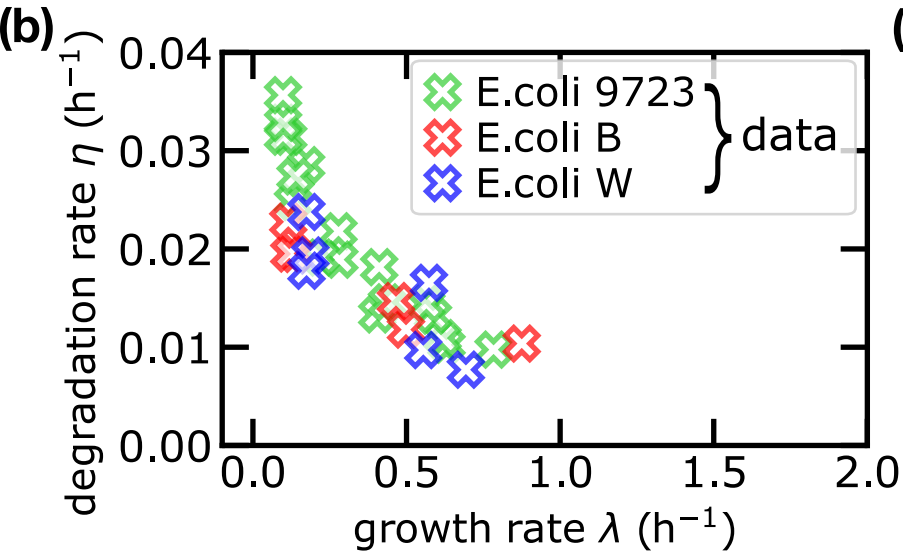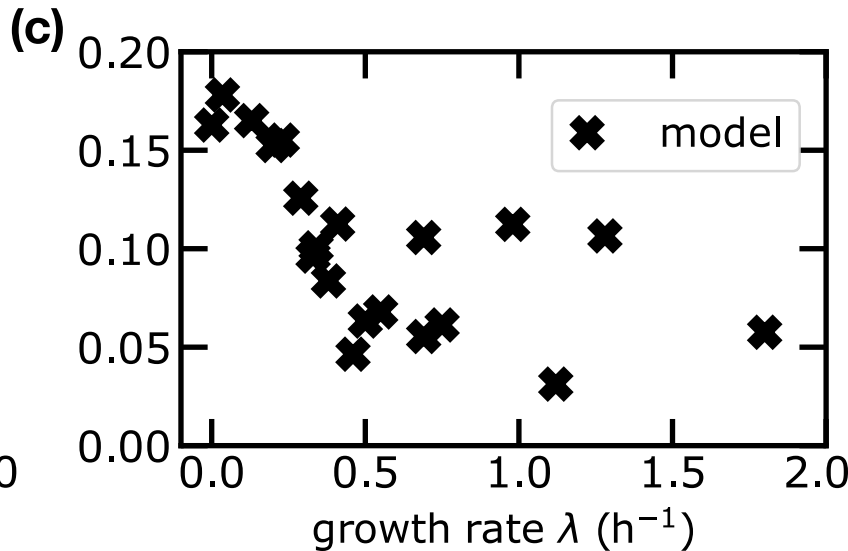

Supplement: S4 Fig — (a) Sketch of the first model of protein production proposed in this work, which includes protein degradation but no inactive ribosomes. In this model, ribosomes follow a first-order kinetics to bind the transcripts, and all bound ribosomes contribute to protein synthesis (mass production). (b) Degradation rate across growth conditions from [38]. (c) Degradation rate estimated from [5] using the model in the first panel. The model captures the qualitative trend of the degradation rate across growth conditions, but fails quantitatively by overestimating the rates by a factor of 4. (PDF) [file pcbi.1010059.s004.pdf]

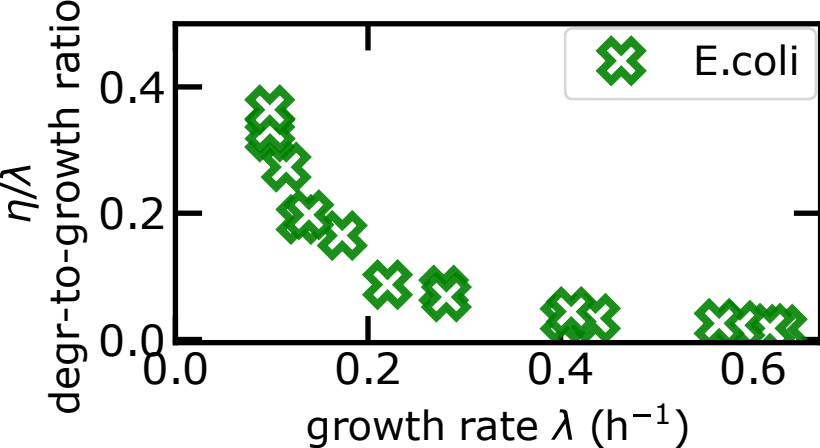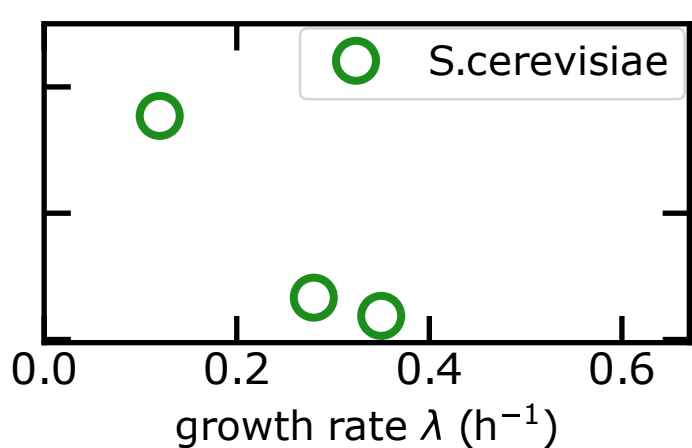

Supplement: S5 Fig — (PDF) [file pcbi.1010059.s005.pdf]

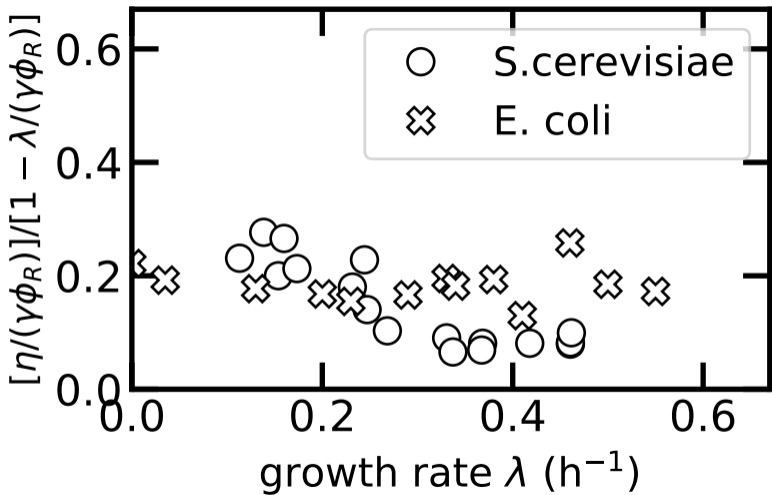

Supplement: S6 Fig — The plot shows that the ratio [η/(γΦR)]/[1 − λ/(γϕR)], evaluated from the available E. coli and S. cerevisiae data (see Methods and materials), is compatible with a constant fb0 ≃ 0.2, across growth conditions, especially for the (much more precise) E. coli data. (PDF) [file pcbi.1010059.s006.pdf]

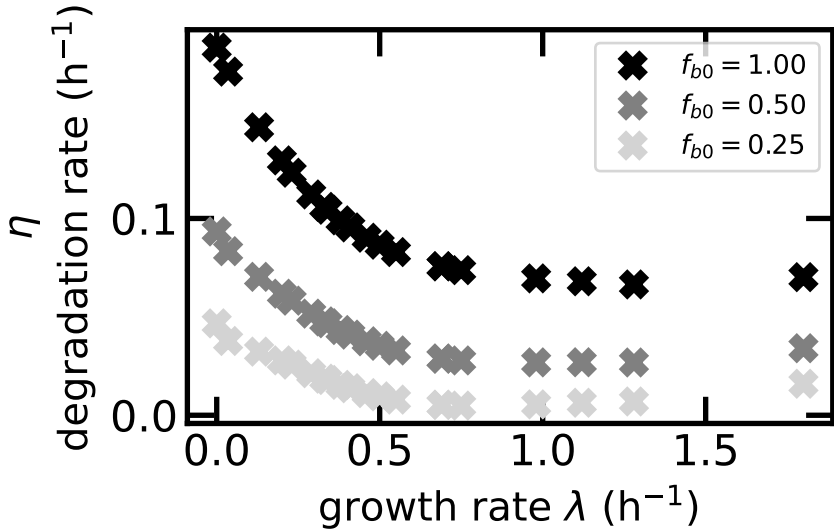

Supplement: S7 Fig — The plot shows degradation rates as predicted by the model equation η = fbϕrγ − λ with fb equal to the constant-ratio ansatz from Eq (14). ϕR and γ are taken from E. coli data given in [5]. (PDF) [file pcbi.1010059.s007.pdf]
