# Supplementary material for: Protein degradation sets the fraction of active ribosomes at vanishing growth
Source: PLoS Comput Biol. 2022 May 2;18(5):e1010059. doi: 10.1371/journal.pcbi.1010059 (PMC9098079; doi:10.1371/journal.pcbi.1010059)
Supplement: Attachment — Submitted filename: Answer_Reviewers.pdf [file pcbi.1010059.s008.pdf]

Portable peer review.

Answer to the Referees for *"Role of protein degradation in growth laws"*, Calabrese et *al.*

This work will be of interest to theorists in microbial systems biology. It shows that taking protein degradation into account improves theoretical predictions of bacterial growth laws at low growth rates. The theoretical aspects of this work are solid. Some underlying assumptions of the model and key predictions remain to be validated experimentally.

We are glad that the referees consider our work to be valuable, and that they view the theory that we developed as a solid contribution.

The two reviewers raise two main points concerning the data from the literature that we have re-analysed or re-used. All these data come from publications by other research groups (either in the recent past or 30-40 years ago).

We answer each of the reviewers' criticisms below. To summarise, they are based on two main points:

(1) inactive ribosomes have never been observed hence testing predictions about their abundance is currently impossible, and (2) protein degradation is important in the limit of zero growth but experimental data in this regime are extremely sparse and challenging (only available for *E. coli*).

Regarding point 1 - Reviewer 1 seems to miss that ribosomes can be inactive through several well-accepted mechanisms (see e.g. PMID: **32649051**), including binding of uncharged tRNAs and being unbound (see e.g. PMID: **20434381**)- inactive ribosomes do not require unspecified active segregation mechanisms. Our theory is agnostic on the origin of inactive ribosomes.

Regarding point 2. We point out that the Dai et al. data are the highest quality available, and form the pillar of several published studies.

Finally, the reviewers' perspective appears to be centered on fast-growing bacteria in the laboratory. However, slow growth is relevant for most of the life cycle of fast-growing bacteria in the wild, for slow-growing bacteria, which are the majority of all bacteria, and for most archaea and eukaryotes. Hence, we believe that there is a strong need for quantitative physiology models able to describe this regime. In addition, many microbial species have maximum growth rates equivalent to the slow growth regime of faster-growing species (e.g. see Kempes et al. 2012, and 2016), and thus this regime has relevance for a diversity of species that grow more slowly than *E. coli* or yeasts. It is important to note that there is significant variation in maximum growth rate across bacterial and single-cell eukaryote species.

Reviewer #1 (Public Review):

Bacteria growth laws are a very interesting field of research with lots of recent activity in trying to understand the older results including that the fraction of a cell that is ribosomes increases linearly with the cell growth rate (regardless of the carbon source). Interestingly, the line doesn't cross the origin at zero growth rate, but has a non-zero offset. This paper aims to address why this is the case and proposes it arises because of the need to devote a pool of ribosomes to maintaining the proteome and compensating for protein degradation, which becomes more important for more slowly growing cells. Yet, while plausible, the data are quite sparse and it is unclear to me how a bacterial cell would have a distinct inactive pool of ribosomes.

Answer: If we correctly understand this remark, Reviewer #1 is discussing the pool of “maintenance ribosomes” that we introduce in this work. However, we do not require unspecified active segregation mechanisms for this pool of actively translating (not inactive) ribosomes, which simply emerges from the balance of the protein production/degradation fluxes. We are stating that a fraction of protein synthesis is needed to replace degraded proteins, and that this is particularly true at slow growth. Via the introduction of maintenance ribosomes, we are able to quantify the fraction of total protein production that is devoted to this task.

The main proposal is that the offset arises because of protein degradation. The hypothesis is that the rate of protein degradation becomes increasingly important at slower growth rates so that to maintain the proteome a larger and larger fraction of ribosomes is engaged in maintenance rather than growth duties. Initially, the degradation rate is considered fixed, but the data gathered from the literature seems to indicate that degradation rates increase at slower growth (Fig. 3). This is pretty interesting as my intuition would have thought that the speed of protein degradation would increase with the cell growth rates since the rates of most processes do. Here, they report the opposite although the data on yeast are pretty sparse.

Answer: We show that a pool of *translating* ribosomes must contribute to the offset and, importantly, that a theory that does not include this contribution is inconsistent at slow growth.

Thus, on the one hand, a model with only protein degradation provides predictions that are not in line with the available data on protein degradation. On the other hand, a model that does not account for protein degradation is also inconsistent with data. We thus conclude that both aspects are necessary to rationalise growth laws in all regimes. Our theory does not aim at understanding the underlying mechanisms behind the origin of active and inactive ribosomal pools, whose existence is well-accepted in the literature.

I'm left wondering about the following major points though:

1. Why don't the authors use the fitted degradation rates as a function of growth rate from Fig 3 for their analysis?

Answer: We did use these data systematically. The reviewer is confused by the storyline, which starts with the falsification of simpler models. We now fixed this problem in the narrative, and we start by clarifying the scope of the degradation-only model and how we use the data in our subsequent analysis:

We will use this simple model in order to falsify the standard view neglecting degradation at slow growth. We will then move to models also including the effects of non-translating ("inactive") ribosomes.
The second part of this study contains a detailed analysis of the available data. As we will see, including degradation is strictly necessary at doubling times that are accessible experimentally in both yeast and bacteria (with high-quality data in E.coli).

2. I don't understand the notion of an inactive pool of ribosomes (eq 12). What evidence is there for the distinction of two separate pools. I could guess that all the ribosomes are infrequently translating protein so that each spends more time unengaged and there appears 'inactive', but it isn't really a separate pool. This assumption seems to me the least compelling, and more data or discussion needs to be brought to bear to justify it.

Answer: We have included these explanations and reference to standard literature on the pool on non-translating ribosomes. Even classic theories describe a reduction of translating ribosomes at slow growth as a decrease of the per-ribosome translation rate (PMID: **1886524**), which is a distinct mechanism from what it is canonically considered to be an *inactive* ribosome (e.g. hibernated ribosomes, see for instance PMID: **32649051**). In *E. coli*, Dai et al measure elongation rates directly, hence it is possible to see in these data how the per-ribosome elongation rate must be the ratio of the physical elongation rate times the fraction of translating (active) ribosomes. Our theory is agnostic on the origin of inactive ribosomes, which is not the focus of our work. Still, arguing on the non-existence of the inactive ribosomal pool seems to be dissonant to well-accepted frameworks, which is not the intent of our work. Instead, we contribute to the open debate about its definition and relevance.

Reviewer #1 (Recommendations for the authors):

As a minor point, I think the boxes are confusing. It would be clearer if the equations were developed in the main text as needed for the arguments. There aren't so many of them so it

would be easy enough to do. And, since the discussion and description of the argument is really mostly in the main text anyway, I don't think the boxes stand all that well on their own.

Answer: Although it is true that there are not so many equations in the manuscript, we believe that integrating the boxes in the text would radically change the organisation of the text. We would rather keep those details in a separate environment for the interested readers only, also to facilitate the reading from a less technical audience.

Reviewer #2 (Public Review):

Bacterial growth laws have enabled considerable progress in our quantitative understanding of cell physiology. The most important growth law describes the dependence of ribosome concentration on growth rate in exponential growth, which is linear with a y-axis offset. In this work, the authors address the origin of this y-axis offset, which is an important conceptual problem. They show that a theoretical model that takes into account both protein degradation and a fraction of inactive ribosomes can explain the empirically observed offset better than the conventional approach, which neglects protein degradation.

Explaining the origin of the y-axis offset in the first growth law would be an important advance with a major impact on the field. The theoretical analysis in this work is carefully performed and the results are clearly presented and easily accessible for a broader audience. However, the experimental support for some key assumptions of the model needs to be clarified and there may be a major conceptual problem with the interpretation of quantities measured at or near growth rate zero.

Specific issues:

1. The limit of zero growth rate, which is the focus of this work, is problematic as key quantities entering the growth laws are not clearly defined in this limit. The authors present an extension of the model first set up by Scott et al. (2010). The original model was designed for cells in steady state exponential growth. At zero growth rate, it is not clear what the steady state is. In exponential growth, this steady state is reached after sufficiently many generations at constant growth rate under constant conditions; however, at zero growth rate, key quantities that are measured will depend on how long the cells were kept at zero growth before the measurement is done (the relaxation time scale of the system becomes infinite in this limit). Very low growth rates with doubling times as long as 10 hours are also hard to detect experimentally and would in practice be treated as zero growth. It should be explained how the measurements at zero growth (from the literature) were performed and how we can be sure that they are as reproducible and clearly defined as those at finite growth rate.

Answer: We point out that the Dai et al. data (PMID: **27941827**) are the highest quality available, and form the pillar of several published studies.

We refer to the methods of that study for details, but all the slow growth points were obtained in controlled steady conditions, and the authors show that they are in agreement with those obtained from sporadic previous studies determined by several different methods. The point at zero growth corresponds to the stationary phase reached in bulk from the 20h interdivision time steady growth.

We have added the following paragraph in the Methods and Materials:

> *A more detailed analysis on E.coli was performed using the Dai et al. data. These data include high-quality direct measurements of translation elongation rates, growth rates, and RNA/protein ratios (ϕ_R), in a wide set of conditions, including slow growth, forming the pillar of several published studies. In this study, all the slow growth points were obtained in controlled steady conditions, and the authors show that they are in agreement with those obtained from sporadic previous studies using several different experimental methods. In this data set, the point at zero growth corresponds to the stationary phase reached in bulk after the steady-growth condition with 20h doubling time.*

2. The authors assume nonspecific degradation in their model. Here, it would be useful to clarify to what extent this assumption holds. I thought that only a small minority of proteins are specifically targeted for degradation in E. coli. A short summary on what is currently known about the common molecular mechanisms of protein degradation in E. coli and S. cerevisiae would be helpful.

Answer: We have added a paragraph describing what is known on degradation in *E. coli*. There is also a nonspecific degradation rate. For the model, what matters is that there is a mean overall degradation dynamics, which impacts growth because ribosomes have to be used to re-translate these proteins rather than translating new ones. We have specified this point in the revised text.

Added paragraph (Introduction):

> *In E. coli, there are many proteolytic enzymes (Maurizi, 1992; Gottesman, 1996).A minority of proteins are specifically targeted for degradation in order to regulate their levels (regulatory degradation), but there also is a basal non-specific degradation(housekeeping degradation), which is important to eliminate damaged or abnormal proteins (Maurizi, 1992; Gottesman, 1996). In yeast, protein degradation has is based on multiple systems that are conserved in eukaryotes up to mammals, such as the proteasome-ubiquitin system (Hochstrasser, 1995) and regulated autophagy (Nakatogawa et al., 2009). Due to this complexity, protein turnover is still not well understood, and remains the subject of current debate (Martin-Perez and Villen, 2017). For our scopes, what will matter is that there is a mean overall protein degradation dynamics; this impacts growth,as biosynthesis will first counterbalance degradation rather than exclusively contributing to a mass net production*

3. Different ranges for protein half-lives are mentioned throughout the paper. The authors acknowledge that degradation time scales between 10 and 100 hours (as mentioned in Goldberg and Dice, 1974; Maurizi, 1992) are negligible (lines 44-46). Later on a simple estimate (lines 90-92) gives degradation time scales of 1-10 hours. However, along with data from Scott et al. (2010) and Metzl-Raz et al. (2017), the authors use their own model to calculate these time scales, more specifically using the assumption that the offset of the ribosomal mass fraction is caused by protein degradation. It needs to be clarified if the degradation time scales needed to explain the offset are consistent with plausible values based on literature knowledge.

Answer: We have revised the coherence of these statements, in order not to confuse the readers. In particular, we made clear the difference of our approaches (model with protein degradation only and model with degradation and inactive ribosomes) and their objectives since the beginning. For instance, we now say at the beginning of the Results section:

> *We start by formulating a simple theory for the first growth law that includes degradation. We will use this simple model in order to falsify the standard view neglecting degradation at slow growth. We will then move to models also including the effects of non-translating ("inactive") ribosomes. The second part of this study contains a detailed analysis of the available data. As we will see, including degradation is strictly necessary at doublingtimes that are accessible experimentally in both yeast and bacteria (with high-quality data in E. coli).*

4. Figure 3 shows results from the final model which includes protein degradation and the distinction between active and inactive ribosomes. In panel b, experimental data for degradation rates is presented and a fit is performed, which is later used to calculate the data points in panel c. The fit for the right plot in panel b includes only three data points and therefore seems arbitrary, especially in the range of 0.4 to 0.6. This is unfortunate as this fit is used for data points that give the crucial comparison between experimental data and the model predictions in panel c.

Answer: The published results on yeast degradation rates are incoherent across studies (see Figure 2-supplement 4a). It would not make sense to attempt a fit across studies and instead we used data from a single study. We chose Gancedo et al. as this is the only study with three measurement points in a wide range of growth rates. We could alternatively have used data from Perez (2017), which are actually less conservative. We have specified this in the text (paragraph after eq.15):

> *We note that the published results on S. cerevisiae degradation rates are incoherent across studies(see again Figure S2.4). Hence, it would not make sense to attempt a fit across studies.Instead, we used data from a single study. We chose data from (J M Gancedo, 1982), this is the only study with three measurement points in a wide range of*

growth rates (from different media). We observe that choosing to use data from (Martin-Perez and Villen, 2017) would increase the prediction of maintenance ribosomes. There is higher coherence for E. coli data. Here, we have chosen again to use data from a single study (Pine, 1973), where the trend is clearest and there are many conditions. Once again other studies report higher degradation rates (see again Figure S2.2), hence the prediction for the fraction of maintenance would increase using values from other studies. Thus, we can conclude that the estimates reported in Fig. 3 have to be regarded as conservative considering existing data.

Figure 3c is quite important for this work, as it captures not only the performance of the model in comparison to the (estimated) data but also the difference between the old model and the new model. The agreement of the lower bound from the model (which corresponds to the case without degradation) appears quite good, especially considering that it has one less free parameter. Here, it would be useful to perform a quantitative comparison of the agreement of the two models with the experimental data to support the relevance of the new model. Additionally, in the legend white symbols are mentioned that are not visible in the plots.

Answer: We have made more explicit the discrepancy between the models in the text that, with the available data, can reach up to 25% for both *S. cerevisiae* and *E. coli*. This information was already present in the text (Fig.4) but it was probably discussed too late.
In the paragraph before the Discussion section, we in fact stated that "*... the fraction of active ribosomes devoted to maintenance f_{bm} as given in Eq. (19) also corresponds to the relative difference $(f_b - f_a)/f_b$.*"
We have added the following paragraph:

> *As it will be detailed in the next section, the relative difference between the model with and without protein degradation (lower bound) depends on the growth rate. It is negligible (a few percent) at fast growth, but we expect it to be larger about 20% when $\lambda \simeq 0.15/h$, and steeply increases to reach 100% when λ approaches zero.*

We removed the reference to white symbols, left from a previous version of the manuscript.

5. Figure 2 - Supplement 2, which shows the degradation rate measured at different growth rates, is crucial for this work and should be a main figure.

Answer: These data are already shown in Figure 3B of the main text.

A discussion of the methods used to measure the degradation rate and an estimate of experimental errors would be helpful. Further, several references for data on degradation rates are given. However, in this figure and throughout the paper only one of these data sets (Pine,

1973) is used for the calculations. Including data of at least one other reference would help to further corroborate the model; it should also be clarified if the different datasets of degradation rates are consistent with each other.

Answer: All these details were already provided in SI. We added more explicit pointers for the interested reader.

It is notable that all references for the degradation rate data are 40-50 years old. The authors mention the methods used for the measurement of these data but it seems necessary to further discuss if these methods are still state of the art.

Answer: This point holds for *E. coli*. As we already wrote in the Discussion, there is an urgency to produce these data (however, ours is a theoretical study). It is unfortunate however that modern yeast SILAC data show higher discrepancies. As a matter of fact, the few SILAC datasets we found show larger estimates of protein degradation rates.

6. In Figure 3 - Supplement 1 experimental data is shown to support the constant-ratio ansatz that is used in the paper. This plot should be corroborated by a quantitative analysis to support the constant behavior of the ratio. For the S. cerevisiae data, it seems from the plot that the ratio is decreasing with increasing growth rate, as the values decrease almost by a factor of five (from ~0.25 to ~0.05). For example, calculating the correlation coefficient and its significance for these data would help to support that they are constant.

Answer: In order to clarify this point, after Eq.15 we have added the following sentences:

> *The agreement is robust with growth rate for E. coli, where precise estimates of elongation rates are available, while for S.cerevisiae the ratio* $\eta/(\gamma\phi_R)$ *decreases for fast growth conditions, but we lack experimental data for the variation of* γ *across growth conditions.*

Reviewer #2 (Recommendations for the authors):

• Figure 2c: Arrow of increasing gamma should point in the opposite direction.
Answer: We thank the reviewer for pointing this out, we have corrected the figure now.

• Figure 2 - Supplement 2: Error bars for degradation rates would be helpful.
Answer: We have added the following sentence in the Methods and Materials section:

> *On the most recent datasets Helbig et al. (2011); Christiano et al. (2014); Martin-Perez and Villén (2017) we estimated the error bars as standard errors of the mean, and they are smaller than the symbols used in the plots.*

In E. coli we could only extract the error bar for the point obtained from Mosteller et al.(1980), see Fig. S2.2. We report the data point η = 0.03/h at λ = 0.52/h from Larrabeeet al. (1980), which is the mean degradation rate estimated from the experiment with the largest number of proteins analysed (359) and following the method explained in that publication. Alternatively, another experiment from the same article would provide a lower bound (as fast-degraded proteins were removed from the analysis) of the mean η = 0.02/h for λ = 0.52/h. However, based on our re-analysis of the data presented in this publication, the error bar we would estimate for this point is almost twofold the mean value and we decided to not report it.

Also, regarding the point from Helbig et al. (2011) we now report the mean value, consistent with the theory, instead of the median.

• Above eq. (2): the assumption that the RNA part of ribosomes does not matter here and just follows the protein part should be explicitly stated.
Answer: We agree with the Reviewer that rRNA also plays an important role (see e.g. PMID: **32701325** or **34389683**), but this should be taken into account in a different equation (and a different model). This framework has been developed to study the role of protein degradation, and there are many other aspects other than rRNA that cannot be considered and are out of the scope of this work (e.g. tRNA, GCN, initiation or elongation factors…).

• line 265-266: The need for including inactive ribosomes (or some other modification of the model) is clear to begin with b/c the protein degradation rate needed without it is way too high (line 92); it would help to state this earlier in the text.
Answer: We have added the following sentence in the paragraph after Eq.2, in order to clarify this point:

These values would correspond to the degradation rates assuming degradation fully explains the observed offset, but they are not distant to the experimental values (~ 10-100 h). This argument suggests that 10% or more of the offset is explained by degradation (see below for a refined estimate).

• Figure 2 - Supplement 3 shows the qualitative agreement between the model predictions with data from Dai et al. (2016) and measured degradation rates from Pine (1973). A quantitative measure for how well these distributions agree with each other would be useful.
Answer: This figure is intended to show the qualitative dependence of the protein degradation rates on growth, that can be obtained with the model accounting for protein degradation only. Instead, showing a quantitative comparison between experimental and estimated data, as suggested by the Reviewer, could be misleading as we use the outcome of the experiments as an input for the full model. Instead, we have better explained in the text the aims of the two models (see also the answers to previous points).
