# Supplementary material for: Protein degradation sets the fraction of active ribosomes at vanishing growth
Source: PLoS Comput Biol. 2022 May 2;18(5):e1010059. doi: 10.1371/journal.pcbi.1010059 (PMC9098079; doi:10.1371/journal.pcbi.1010059)
Supplement: Attachment — Submitted filename: Response_to_reviewers.pdf [file pcbi.1010059.s009.pdf]

Dear Editor,

We thank you and the referees for the detailed reports.

We have modified the manuscript according to the recommendation and followed the advice of referees to simplify the narrative.

Please find below the point-by-point replies to the 3 reviewers.

Below we briefly summarize the changes made, the details can be found in our reply and in the pdf of the revised article with the highlighted changes.

Yours sincerely,

The authors

SUMMARY OF CHANGES

- As suggested, we have changed the title. The new one reads: “Protein degradation sets the fraction of active ribosomes at vanishing growth”.
- We have simplified the narrative and we now first explain the current ‘standard’ framework (inactive ribosomes and no degradation), then introduce a model with protein degradation only, and finally show how combining inactive ribosomes and degradation best explains the data. This implied reversing the order of Box 1 and 2 and moving a supplementary figure to the main text (now Fig.2). The table with the symbols used in the text has also been updated with including relevant values for *E.coli* and *S.cerevisiae* and it has been moved at the beginning of the manuscript.
- We clarified the different possible contributions to the inactive ribosomes pool.
- The emphasis on the role of maintenance ribosomes has been reduced and we no longer mention them in the Author’s summary, making it clear that this distinction is not functional.
- We corrected typos and clarified estimates provided in the previous version of the manuscript, better highlighting sources when necessary.

REPLY TO THE REVIEWERS

Reviewer #1: The authors have thoroughly addressed the issues I had raised in my previous report. These points are now much clearer. I support publication of the manuscript.

I have one remaining suggestion: The authors should consider adding a figure (or modifying the current figures) to more clearly highlight the main advantage of the new model, which accounts for degradation, in explaining experimental observations. This is still a bit hidden in Figure 3.

We thank the reviewer for the work on our manuscript and for the positive remarks. We have followed their advice and modified Fig. 3 accordingly: we modified the label 'lower bound' to explicitly reference the model without degradation; with the same color code draw a line for zero degradation in panel (b); added Figure S5 to show the regimes in which degradation becomes comparable with growth; Better explained in the text the comparison between models with and without degradation (see also answer to Reviewer #2).

Reviewer #2: Many microbes spend much of their life in slow growth, yet our quantitative understanding of microbial physiology (in terms of growth laws) is so far limited to medium and fast growth. As such Calabrese et al make a valid contribution by highlighting the importance of protein degradation and ribosome activity to improve current growth theories.

They do so by analysing three versions of a simple steady-state growth model, with and without degradation (but with inactive ribosomes), and with degradation plus inclusion of inactive ribosome, and find that only the latter satisfactorily explains combined datasets of previously published growth rates, protein mass fractions and translation elongation rates from E. coli and S. cerevisiae. Data limitations are in my view satisfactorily discussed. The analysis is solid, however, it hinges on the assumption of steady-state, as pointed out by another reviewer. It is questionable whether very slowly growing cells are ever in steady state, as adaptive processes are likely to be ongoing. It could be that this is negligible, given the macroscopic level of description of growth used in this study, but it should be mentioned in the discussion or elsewhere that this is a potentially limiting assumption.

The paper is well written, and my comments are minor. Among them, the most major ones concern the title and last section of the paper.

We are grateful to the reviewer for the positive remarks and for the work on our manuscript.

Title "Role of protein degradation in growth laws": the title should be more specific, in particular, as only one growth law is considered in this paper.

We agree with the referee and changed the title into *Protein degradation sets the fraction of active ribosomes at vanishing growth.*

Section "The fraction of active ribosome increases with protein degradation to the added presence of ribosome devoted to maintenance":

The section, including Fig 4, add little except stating the obvious that, if synthesis needs to compensate for degradation and degradation is non-neglible at slow growth, a sizeable fraction of active ribosomes will be involved in maintenance. Also the classification between 'growth'- and 'maintenance'-ribosomes seems rather odd, as if they had different functions. I suggest that the authors stick to their original definition of growth rate as the surplus of protein synthesis over degradation, implying that at zero growth all synthesis replaces degraded proteins and thus can be considered maintenance.

We agree that the main results are already provided in the previous section. However, we believe that this section is important to better compare the outcome of our model to the standard approach neglecting protein degradation. We revised the text to meet the Reviewer’s point. We now reduced the emphasis on the role of maintenance ribosomes (which are no longer mentioned in the Author’s summary), making it clear that this distinction is not functional.

Further minor comments:

- After Eq (15), the text seems to refer to an outdated version of Fig S3.1, as this is not a scatter plot of the two terms considered, where a slope should be extracted, but rather the ratio of the two terms across different growth rates, which the authors conclude is near-constant.

- The lines in Fig. 3b are not labelled.

- I suggest that Table 1 is included as another box at the beginning of the paper (before Box 1). This would improve readability, as throughout the manuscript, parameters are often only referenced by their variable name and not by their meaning, which makes for a lot of scrolling and searching for definitions. It would also help, as the boxes are not self-contained with regard to parameter definitions.

We have fixed these problems. Regarding the labeling of Fig3b, due to the lack of space we added a sentence in the caption. We followed the reviewer’s advice and moved Table 1 at the end of the first Results section.

- Finally, I appreciate that this might not be minor work at this stage of the manuscript, hence I am including this as a suggestion only: The logical flow of the paper could benefit if the order of models were rearranged: (1) starting with the current 'standard' assumption of inactive ribosomes and no degradation (currently Box 2) and how this theory fails at slow growth, Eq (11); (2) asking if a degradation model (currently Box 1) could explain the y-offset instead; (3) the combination of inactive ribosomes and degradation best explains current data (currently Box 3).

We have followed the reviewer's advice and inverted the order, which also addresses the main issue raised by Reviewer three.

- Some typos:

> 2nd last paragraph of intro: "In yeast, protein degradation (has) is..."

> Box 1: "Since initiation is about two orders of magnitude slower..." rather than "order of magnitudes"

> Missing punctuation in the paragraph above Eq (21)

We fixed the typos, and thank the reviewer for pointing them out.

Reviewer #3: Growth laws have been developed primarily for fast growth of unicellular organisms, i.e. when nutrients are plentiful. The current manuscript focuses on a different regime, in the limit of zero growth rate, when the cell cycle time is prolonged and becomes comparable to typical protein lifetimes. These are extremely slow growth rates (bordering on no growth, i.e. stationary phase).

The authors argue that protein degradation is significant at slow growth rates and to counter it requires "maintenance" ribosomes. They attribute the offset (i.e. non-zero intercept of ribosomal proteome fractions vs growth rate) to protein degradation. It is difficult to judge the merit of the proposed hypothesis as there is simply insufficient data to verify it. Furthermore it is still not clear whether protein degradation is a significant effect based on the evidence presented – the authors themselves note that only ~10% of the offset is explained by degradation (see top of p.5). I therefore recommend that the claims not be over-stated, such as in the last sentence of 'Author summary'. It should be emphasized that it has not been definitively shown that protein degradation is the primary cause of the maintenance ribosome fraction.

We have updated the last sentence of the author summary to tone down the statement. It is important to clarify that a model without degradation is clearly ruled out, especially with the (better-quality) *E. coli* data. We modified Fig 3 and revised the text, which now should describe more clearly the differences between the two models. The revised manuscript highlights the advantages of a model including degradation, as also suggested by reviewer 1. Finally, we have also stressed in the revised text that with a first back-of-the-envelope calculation we obtain a 10% of the offset is explained by degradation, but that a refined estimate (our model, considering precise *E. coli* data from Dai et al. 2016) leads to an estimate of ~20-25\% of active ribosomes contributing to the offset at zero growth.

Furthermore, the models in the manuscript do not account for regulatory feedback mechanisms which may come into play at low growth rates, as such growth rates correspond to stressful conditions. The latter point should be emphasized.

We agree with this point, which is part of our current plans. We added a comment in the Discussion section.

Some statements in the manuscript appear inaccurate. For example, in the second paragraph of the introduction, Klumpp et al. 2013 showed a growth-rate dependent translation rate in E. coli (not active ribosome fraction, see their Fig.1B). Furthermore, there are methods of experimentally estimating ribosome activity, such as polysome profiling (see, e.g. Metzl-Raz 2017). Also, the non-zero ribosomal proteome fraction at low growth rates is usually interpreted in the literature as a ‘reserve’ fraction for the cell, to prepare for nutrient upshift (e.g. Metzl-Raz 2017). The nutrient upshift hypothesis should be added to the discussion.

We removed the reference to Klumpp as the main study is Dai et al. (it was intended as related to that study).

We added the nutrient upshift hypothesis in the introduction and in the discussion section.

In order to clarify the different statements about the experimental identification of active ribosomes, we added a few sentences in the text to explain how inactive ribosomes have been defined in the literature (for instance in Dai et al. 2016 and Dai&Zhu 2020, and Wu et al. 2021). We now mention for instance that:

… ribosomes can be inactive for different reasons (e.g. ribosomal subunits sequestered in the cytoplasm, ribosomes blocked in traffic, or carrying uncharged tRNAs),

or

in the standard interpretation, ribosomes can be inactive because they are sequestered for different reasons (even inside a translating mRNA)...

In general, we are not aware of a method to *directly measure* (we added this in the text) the fraction of sequestered/inactive ribosomes. The method provided in Metzl-Raz 2017 is based on polysome profiling, which cannot distinguish between active and inactive/sequestered ribosomes, considers 80S ribosomes as inactive (it is a debated point in the literature) and for which the choice of considering the sum of 40S+60S subunits as inactive ribosomes remains arbitrary. We now explicitly mention this approach in the introduction highlighting the first problem.

To summarize, we believe we clarified these points in the introduction and the discussion. A central point is that there are at least two sources of inactive ribosomes, one related to uncharged tRNAs and affecting translation rates, one is putative active sequestration mechanisms (cite Wu et al. 2021). The literature is ambiguous in these regards, but we now clarified this point.

The narrative of the paper is convoluted and hard to follow, as the authors go back and forth between models with/without active ribosomes. I recommend streamlining the narrative to make it easier for the reader to comprehend the main points.

We have followed the specific advice from reviewer 2 and inverted the order, which should address this point.

Specific remarks:

Please revise the introduction (particularly second paragraph) according to the comments above.

We have edited this paragraph accordingly.

1st paragraph of results section, last sentence – please explicitly state the experimentally accessible doubling times for E. coli and yeast where protein degradation becomes ‘strictly necessary.’

We added this information as a comment to Fig. S5

Paragraph above Eq. 2: Is there evidence supporting the assumption that protein degradation rates are constant across growth rates, as assumed? Also, please provide the reader with values of k and L_R for E.coli/yeast when defining gamma := k/L_R.

We only have assumed constant degradation rates for introducing the model, and then considered degradation rates across growth rates for *E. coli*, where data are available. We have clarified this point in the text. We have also added in Table 1 the values of parameters for *E.coli* and yeast.

Last sentence on p. 4: It would be helpful for the reader if the authors explain how they arrive at gamma ~3.6 to 7.2 hr^(-1) and 1/eta ~1 to 10 hr. My calculation yielded the following: For E. coli (using Scott et al. data for a minimum value of RNA-to-protein ratio of 0.1, which corresponds to phi_{R}^{min} = 0.076), L_R = 7536 and k ranges from 13 to 22 a.a./sec (see Bremer & Dennis), yielding gamma of 6.2 to 10.5 hr^(-1) and 1/eta of 1.25 to 2 hr.

We have clarified this point. We have used the value for k directly measured by Dai et al 2016 (now cited here). Hence, by considering k = 8 aa/s we obtain gamma of about 4/h (and 1/eta~ 5 h in *E.coli*). We have also corrected the typo in the range of phi_{R}^{min} (previously 0.02-1) and given the values 0.05-0.08 from Dai et al. 2016 and Metzl-Raz et al. 2017.

Fig. 1. Add tickmarks on the axes so the reader has a better feel for the relevant growth rates. The plot in Dai et al. indicates deviations from the growth law for lambda < 0.6 hr^-1 and RNA-to-protein ratio of 0.1 (corresponding to ribosomal protein mass fraction 0.076).

We think this is not necessary as this figure is generic and purely illustrative. The values of the parameters will be organism-dependent.

Box 1. Many parameters defined but hardly any characteristic values are provided for rho, tau_e, etc.

We have now added relevant values in Table 1.

Above Eq. 7 the authors write “It is yet experimentally unfeasible to distinguish between active and inactive ribosomes (Zhu et al., 2020).” I could not find such a conclusion in the reference cited. Could the authors please clarify? Ribosome activity has been estimated by experimental means, e.g. polysome profiling, in Metzl-Raz et al. 2017, so the authors’ statement appears inconsistent with the literature.

This is related to a previous point. We now explain that there are different sources of inactive ribosomes, one related to uncharged tRNAs and affecting translation rates, one is putative active sequestration mechanisms. Zhu et al. explicitly discuss the sequestration hypothesis, and comment that in *E. coli*, no proteins that perform this task have been identified. We have made this more clear in the revised text. Recent papers (e.g. Wu et al bioRxiv 2021, Hwa group) start including both sources of active ribosome separately.

We now refer to the approach proposed in Metzl-Raz et al. 2017, as discussed above.

Below Eq. 7: Could the authors comment on how their definition of ‘inactive’ ribosomes differs from other definitions of activity in the literature? What is the physical origin of their ‘inactive’ ribosomes if they are not included in the pool of non-translating cytoplasmic ribosomes?

In the active ribosomes model we have defined inactive ribosomes following the work by Dai and coworkers (Dai 2016), which does not identify cytoplasmic or bound ribosomes. In other words, we are using as a reference the most recent model in the literature, which has been compared to precise measurements.

In our model we have defined bound ribosomes as active, thereby describing the role of uncharged tRNAs as changes in translation elongation rate.

On the interpretation side, see the considerations above (clarified in the revised text).

I have difficulty following the authors’ arguments after Eq. 11. The product of ribosome activity and elongation rate follows a Michaelis-Menten behavior on growth rate for E. coli and yeast (Klumpp et al. PNAS 2013; Kostinski & Reuveni Phys. Rev. Res. 2021). How does that fit into the authors’ arguments regarding Eq. 11 and protein degradation?

The point is that for lambda->0 , as shown by Dai and coworkers, the slope of the first growth law changes - so the MIchaelis Menten approximation used in these studies is valid only for sufficiently high growth rates. This picture is inconsistent with protein synthesis at vanishing growth. We included a sentence to clarify that even the fact that the product of ribosome activity and elongation rate follows a Michaelis-Menten behavior on growth rate is inconsistent with the observation of protein synthesis at vanishing growth.

Fig. 3c: The authors deduce that the active ribosome fraction increases with growth rate, however Bremer & Dennis report that this fraction stays constant at 85% in E. coli for different growth rates. Could the authors please comment on this discrepancy?

This analysis fully follows the work by Dai and coworkers (Dai et al 2016), in which they show that the fraction of active ribosomes is ~0.8 at fast growth, but drops for growth rates smaller than ~0.5/h.

How did the authors arrive at Eq. 14 (the ansatz)? The data plotted in Figure S3.1 appears to vary quite a bit from 0.2 for S. cerevisiae; can the authors please quantify the variation from 0.2, i.e. relative error.

This ansatz emerged from the analysis of the (better quality and more comprehensive) *E. coli* data, and we applied it to both systems. The current yeast data show two regimes (probably because no relation k(lambda) has been measured for this organism and we considered a constant gamma), but better data would be required to fully test this ansatz.

p. 12, last two sentences: I could not follow the argument regarding ‘the most direct evidence for non-translating ribosomes’ and how it leads to a ‘dark matter’ problem. I was under the impression that it has long been known that not all ribosomes in the cell are active. Please expand and clarify.

This again has to do with the ambiguous definition of inactive ribosomes in the literature. We did our best to clarify that in the text and in this reply.

Table 1: I would suggest adding typical values for the parameters listed, to be easily accessible to the reader.

We have included a list of values in Table 1.
